# Contributions to the Study of the Heart and Lungs

**Published:** 1888-03

**Authors:** 


CONTRIBUTIONS TO THE STUDY OF THE HEART AND LUNGS. By James R. Leaming, M. D. E. B. Treat & Co.; price, $2.75. New York, 1887.

It must require some courage on the part of an author who writes a book on the diseases of the chest, so numerous are the works in this branch of medical inquiry. This work of Dr. Leaming's is composed of a series of essays read or published at different times; they are well written, and seek to elaborate some disputed points in the diagnosis and the pathology of chest diseases.

